# Heavy Metal Pollution and Health Risk Assessment of Vegetable–Soil Systems of Facilities Irrigated with Wastewater in Northern China

**DOI:** 10.3390/ijerph19169835

**Published:** 2022-08-10

**Authors:** Zhe Xu, Mingyi Shi, Xiaoman Yu, Mingda Liu

**Affiliations:** 1 College of Land and Environment, Shenyang Agricultural University, Shenyang 110866, China; 2 School of Geographic and Environmental Science, Tianjin Normal University, Tianjin 300387, China; 3 Key Laboratory of Industrial Ecology and Environmental Engineering (Ministry of Education, China), School of Environmental Science and Technology, Dalian University of Technology, Dalian 116024, China

**Keywords:** heavy metals, facility vegetables, soil, waste water irrigation, health risk assessment, hazard index

## Abstract

Soil pollution by heavy metals is a major concern in China and has received much attention in recent years. Aiming to investigate the status of heavy metal pollution and the safety of vegetables in the soil of wastewater-irrigated facilities, this study investigated the distribution and migration characteristics of heavy metals in vegetable–soil systems of facilities in a typical sewage irrigation area of the Xi River, Shenyang City, northern China. Health risks due to the fact of exposure to heavy metals in the vegetable soil of facilities and ingrown vegetables through different exposure pathways were evaluated. Spatial interpolation and a potential ecological risk assessment were applied to evaluate the soil quality. Bioaccumulation factors (BCFs) were used to analyze the absorption and transportation capacity of Cd, Cu, Pb, and Zn by different parts of different vegetables. The results showed that the average concentration of Cd exceeded the standard values by 1.82 times and accumulated by 11 times, suggesting that Cd poses the most severe pollution among the four metals in the soil of facilities in the Xi River sewage irrigation area. In the city, a significant accumulation of Cd in the soil was identified with different spatial distributions. Cd also contributed the most in terms of the estimated potential ecological risk index, while the impacts of the other three metals were relatively small. The concentrations of heavy metals were mostly lower than the limit set by the corresponding Chinese standards. Various BCFs were observed for the four metals in the order Cd > Zn > Cu > Pb. Vegetables also demonstrated different BCFs in the order of leaf vegetables > Rhizome vegetable > Solanaceae vegetable. The magnitude of the noncarcinogenic risk for all four heavy metals was less than one for all three exposure routes and did not cause significant noncarcinogenic health effects in humans. However, the carcinogenic risk of Cd from some vegetables via dietary intake was considered higher. Protection measures should be taken to implement better pollution control and land use planning.

## 1. Introduction

China is the largest vegetable producing country in the world, accounting for 36% of the world’s vegetable planting area and 66% of the world’s vegetable production [1]. With China’s economic and social development, the standard of quality for vegetables has increased. Hence, heavy metal pollution in vegetables has drawn great attention nationwide. Rapid development of facilities in the vegetable industry has occurred, and research on the accumulation of heavy metals in these lands has gradually increased. Several studies have suggested that the decline in soil quality was caused by the increase in the level of heavy metals, which was the result of the excessive usage of vegetable land, fertilizer, and a high cropping index [2,3,4,5]. Studies by several groups have also reported the transfer of pollutants from the soil into the food chain, endangering residents’ health [6,7,8,9]. The issue of declining soil quality is related to metal pollution in sewage irrigation areas, which has attracted many researchers’ attention due to the accumulation of heavy metals, which is hazardous to food safety and human health [10,11,12,13,14,15]. Hence, it is necessary to perform investigations on heavy metal pollution, as monitoring, prevention, and controls are needed to ensure the quality and safety of the vegetables.

Differences in bioaccumulation ability can be found among different crops. In general, the abilities of absorption, conversion, and bioaccumulation of heavy metals in vegetables are higher than that in traditional plant crops. Some vegetables can not only absorb some heavy metals but also have special organs with the bioaccumulation ability to accumulate pollutants including As in the roots of carrot, Hg in bean pods, Pb and Cd in the roots of radish, and Se in the leaves of radish [16]. The concentration of heavy metals in soil generally increases over time in sewage irrigation areas. All of this results in the risk of the bioaccumulation of heavy metals by the vegetables planted in sewage irrigation areas. Due to the fact of accelerated urbanization and continuous increase in planting area and the production of vegetables in northern China, the issue regarding heavy metals in the soil in these areas has become prominent [1,17].

The Xi River sewage irrigation area in Shenyang, northern China, is an atypical irrigation area that was irrigated by industrial wastewater from the 1950s to the 1990s. It is one of the areas irrigated for the longest continuous period, with the most serious pollution in China [18,19,20]. Although there has been no continuous sewage irrigation in that area for more than twenty years, and the local government has implemented policies, including adjusting the planting crops, the soil and crops are still affected by the sustained effects. The potential ecological risks from the irrigation of accumulated sewage warrant continuous monitoring and investigations, as heavy metals are difficult to degrade and remove from the soil. In addition, whether the risk to human health has been altered due to the fact of changes in people’s diet is unknown. Meanwhile, researchers have found that despite the heavy pollution, the Xi River water is still used as a source of agricultural irrigation due to the lack of clean water [20]. Xi River water has greatly accumulated heavy metals, as it has been polluted by sewage from industrial and agricultural use in addition to the surrounding cities. Different from a previous study on the farmland soil of the Xi River sewage irrigation area in Shenyang [21], this study took facilities’ vegetable soil as the research object. There is a certain difference between the two farming and irrigation methods. The accumulation and spatial distribution characteristics of heavy metals in soil were investigated to evaluate the risks to the local communities’ health [22,23,24]. Aimed at investigating the heavy metal pollution status and vegetable safety in the soil of wastewater-irrigated facilities, this study investigated the distribution and migration characteristics of heavy metals in a facility’s vegetable soil–vegetable system in a typical sewage irrigation area of the Xi River, Shenyang city, northern China. Meanwhile, health risks due to the fact of exposure to the heavy metals in the facility’s vegetable soil and the grown vegetables through different exposure pathways were evaluated to provide scientific data to support the adjustment of the spatial arrangement for vegetable food safety, the agricultural planting approach, and the land-use planning in that region.

## 2. Materials and Methods

### 2.1. Sample Collection and Analysis

**Figure 1:**
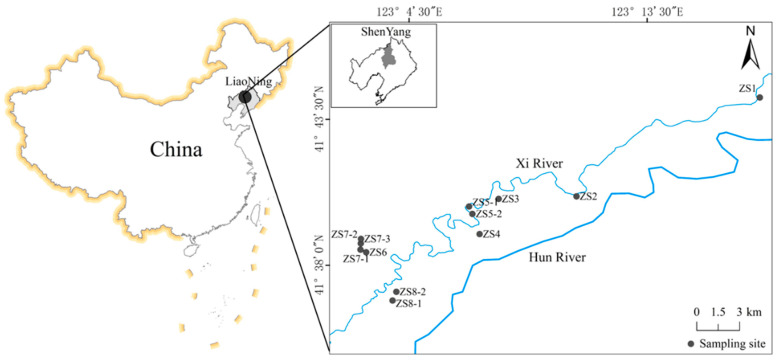
Geographical locations of the sampling sites in the study area.

The Xi River sewage irrigation area is located in west Shenyang (41°36’~41°45’N, 123°02’~123°18’E), and it is a typical sewage irrigation area in northern China. Wastewater and domestic sewage in the industrial zone of west Shenyang were used as irrigation in this area after being diluted with a small amount of Hun River water. As a tributary of the Hun River, the Xi River originated in the Tiexi District of Shenyang city, with a length of approximately 80 km and an average width of 8 m [19]. In this study, the sampling points were distributed along the river basin (Figure 1).

In April 2019, 0–20 cm layer of arable soil and vegetable samples were collected. A total of 12 sampling points in the greenhouse area of typical facilities were set-up, and each sample consisted of five equiponderance subsamples taken from the surface of five sites. The GPS locations for the sampling sites are shown in Figure 1. After the soil samples were evenly mixed, each one was reduced to 100 g by the quartering method. Then, every sample was dried in an oven, with the tiny stones, animals, and plant residues in the samples removed. All samples were evenly mixed again after being ground using an agate mortar and passed a 0.149 mm nylon sieve (100 mesh). There were 35 vegetable samples, including 8 leafy vegetables (i.e., 1 cabbage, 4 coriander, 1 chlorella, 9 lettuce, 3 Chinese lettuce, 3 asparagus leaves, 2 celery, and 2 spinach),1 rootstock vegetable (i.e., 7 radishes), and 1 solanaceous vegetable (i.e., 3 tomatoes), collected from the study area. Vegetable samples were washed with tap water and purified water to remove the dirt and soil grains and then sterilized at 105 °C and dried at 65 °C. Fresh and dry weights were recorded before and after the drying process. Dried samples were further ground to pass through a2 mm sieve and stored in plastic bags at room temperature until analysis.

Prior to the measurement of the metal contents, the soil samples were digested with HNO_3_-HF-HClO_4_, and the vegetable samples were boiled with HNO_3_. Inductively coupled plasma was used to measure the concentrations of Cd, Cu, Pb, and Zn in the soil and vegetable samples. Parallel samples and blank samples were included for quality control purposes to evaluate the reliability and accuracy of the measurements.

### 2.2. Risk Calculations

#### 2.2.1. Heavy Metal Ecological Risk Index

The Potential Ecological Risk Index method was developed by the Swedish scientist Hakanson to quantitatively evaluate the extent of heavy metal contamination in sediments [25]. The method takes into account the heavy metal content, ecological effects and environmental toxicological characteristics at the same time and is calculated by the formula:

(1) $$\mathrm{RI}=\overset{m}{\underset{i=1}{\sum }}{\mathrm{Er}}^{i}=\overset{m}{\underset{i=1}{\sum }}{\mathrm{Tr}}^{i}{\times C}_{f}^{i}=\overset{m}{\underset{i=1}{\sum }}{\mathrm{Tr}}^{i}\times \frac{C^{i}}{C_{r}^{i}}$$

where i represents the names of heavy metals; RI refers to comprehensive potential ecological risk factor; ${\mathrm{Er}}^{i}$ is the potential ecological risk factor of substance i; ${\mathrm{Tr}}^{i}$ is the toxic reaction factor of element i in the reference value from the toxic factors of Hakanson’s research (the values for Cd, Cu, Pb, and Zn are 30, 5, 5, and 1, respectively) [25]; $C_{f}^{i}$ is the pollution factor of a single substance i; $C^{i}$ is the tested concentration of substance i in mg·kg^−1^; m is the number of heavy metals; $C_{r}^{i}$ is the reference value of element i for comparison, where the background values of heavy metals in Liaoning Province were used. The results were grouped into five classes, namely, low risk (${\mathrm{Er}}^{i}$ < 40, RI < 150), moderate risk (40 ≤ ${\mathrm{Er}}^{i}$ < 80, 150 ≤ RI < 300), considerable risk (80 ≤ ${\mathrm{Er}}^{i}$ < 160, 300 ≤ RI < 600), high risk (160 ≤ ${\mathrm{Er}}^{i}$ < 320, RI ≥ 600), and very high risk (${\mathrm{Er}}^{i}$ ≥ 320) [25].

#### 2.2.2. Vegetables’ Bioaccumulation Index for Heavy Metals

The vegetables’ bioaccumulation index for heavy metals was used to measure the ability of vegetables to absorb and accumulate heavy metals [26]. BCFs were calculated using the following equation:

(2) $$\mathrm{BCF}=\frac{C_{vegetable}}{C_{soil}}$$

where $C_{vegetable}$ is the concentration of heavy metals in the edible parts of the vegetables (dry weight, mg·kg^−1^); $C_{soil}$ is the total concentration of heavy metals in the soil.

#### 2.2.3. Health Risk Assessment

There are four human exposure pathways for heavy metals in soil. The first one is dietary ingestion through the food chain, where heavy metals are transferred into human bodies through fruits, vegetables, and crops grown in polluted soil. The second one is the direct ingestion of soil grains. The third one is the inhalation of polluted soil dust via the mouth or nose. The fourth one is dermal intake, where skin directly contacts polluted soil particles. Formulas for the calculation of the health risk, proposed by the US Environmental Protection Agency in combination with other studies [3,21,27], were used to evaluate the risk levels of heavy metals in the soil through dietary ingestion. Health risks can be divided into carcinogenic and noncarcinogenic risks. Noncarcinogenic elements in this study included Cd, Pb, Cu, and Zn, with Cd also being a carcinogenic element [28]. Cd is classified as a confirmed carcinogen for human lung and prostate cancers by the International Agency for Research on Cancer, and it can also cause multiorgan damage, mainly in kidney and bone, cardiovascular and cerebrovascular diseases, and metabolic diseases [29,30,31].

The daily exposure doses of contaminants via the various exposure pathways were calculated using the following equations:

(3) $${\mathrm{ADI}}_{dietary}=\frac{C_{vegetable}\times I_{intake}\times \mathrm{EF}\times \mathrm{ED}}{\mathrm{BW}\times \mathrm{AT}}$$

(4) $${\mathrm{ADI}}_{ing}=\frac{C_{soil}\times \mathrm{IngR}\times \mathrm{EF}\times \mathrm{ED}\times \mathrm{CF}}{\mathrm{BW}\times \mathrm{AT}}$$

(5) $${\mathrm{ADI}}_{inh}=\frac{C_{soil}\times \mathrm{InhR}\times \mathrm{EF}\times \mathrm{ED}}{\mathrm{BW}\times \mathrm{AT}\times \mathrm{PEF}}$$

(6) $${\mathrm{ADI}}_{dermal}=\frac{C_{soil}\times \mathrm{SA}\times \mathrm{AF}\times \mathrm{ABS}\times \mathrm{EF}\times \mathrm{ED}\times \mathrm{CF}}{\mathrm{BW}\times \mathrm{AT}}$$

where ADI is the average daily intake of heavy metals in vegetables (mg·kg^−1^·day^−1^); C is the tested concentration of element i in the sampling area (mg·kg^−1^). All parameters and their definition are summarized in Table 1.

(1) Noncarcinogenic risk assessment
  (7) $$\mathrm{HQ}=\frac{\mathrm{ADI}}{\mathrm{RFD}}\;\;\mathrm{HI}=\sum^{\;}\mathrm{HQ}$$
  where HQ is the ratio of exposure to a single noncarcinogenic substance; HI is the total risk index to the noncarcinogenic composition of multiple elements; RFD is the noncarcinogenic reference dose of a contaminant (mg·kg^−1^·day^−1^). The ${\mathrm{RFD}}_{dietary}$ values for Cd, Cu, Pb, and Zn were 0.001, 0.037, 0.0035, and 0.3 mg·kg^−1^·day^−1^; ${\mathrm{RFD}}_{inhalation}$ values for Cd, Cu, Pb, and Zn were 0.001, 0.0402, 0.00352, and 0.3 mg·kg^−1^·day^−1^; ${\mathrm{RFD}}_{deamal}$ values for Cd, Cu, Pb, and Zn were 0.005, 0.0019, 0.00552, and 0.06 mg·kg^−1^·day^−1^ [32,33,34,35].
(2) Carcinogenic risk assessment
  (8) $$\mathrm{CRI}=\mathrm{ADI}\times \mathrm{SF}\;\;\mathrm{RT}=\sum^{\;}\mathrm{RI}$$
  where CRI is the probability of health risks from a carcinogen to an individual; SF is the carcinogenic slope factor of a carcinogenic contaminant (mg·kg^−1^·day^−1^). We only included the risk of cancer from dietary intake (SF = 6.1) and inhalation (SF = 6.4) in this study. RT is the cumulative cancer risk of the composition of multiple heavy metals. The only carcinogenic element was Cd in this study.

### 2.3. Data Analysis

Descriptive statistics were calculated using SPSS 19. Origin 8 was employed for the graph plotting. Spatial distribution mapping of heavy metal concentrations using ordinary Kriging was performed with ArcGIS 10.6 (ESRI Co., Redlands, CA, USA).

## 3. Results and Discussion

### 3.1. Heavy Metals in Soil

#### 3.1.1. Concentrations of Heavy Metals in Soil

The concentrations of heavy metals, in general, exceeded the standards in the greenhouse soil of the Zhangshi sewage irrigation area (Table 2). Compared with the “*Environmental Quality Evaluation Standard for Farmland of Greenhouse Vegetable Production (HJ333-2006)*” (pH < 6.5) [36], the concentrations of Cd, Cu, Pb, and Zn were 0.66–3.02 times, 0.3–1.77 times, 0.35–0.67 times, and 0.41–1.26 times that of the corresponding standard values, respectively, where the average concentration of Cd and Cu exceeded the standard values by 1.82 and 1.01 times. Compared with the soil background values in Liaoning Province [37], the four metals had different accumulations, where Cd, Cu, Pb, and Zn had accumulated by 3.95–18.14, 1.54–8.98, 1.73–3.27, and 2.88–8.92 times, respectively. The heavy metal cadmium (Cd) was found to be one of the important pollutants in the Xi River sewage irrigation area in this study, which is consistent with previous studies [18,21]. The coefficient of variation (CV) is regarded as an important indicator of the degree of interference of soil heavy metal content distribution by human activities. The degree of variability can be classified as weak variability (CV < 10%), moderate variability (10% ≤ CV < 100%), and strong variability (CV > 100%) [38,39]. The coefficients of variation (CV) for Cd and Cu were 40.84% and 46.65%, respectively, and they were highly variable (CV > 36%); the CV of Pb and Zn were 20.59% and 27.00%, respectively, and they were moderately variable (15% < CV < 36%). These data show that the concentrations of cadmium and copper varied between samples from different locations, while the distributions of Zn and Pb were relatively even with smaller differences.

Compared to existing studies over the last 20 years [40,41,42], there was a significant decline in the levels of Cu and Zn in the soil, while no decrease in Cd and Pb in the soil was observed, even after an extended period of recovery from sewage irrigation. In comparison with similar sewage irrigation areas in other cities (i.e., Tianjin and Beijing), the contents of Cd and Pb in the sewage irrigation soil in Shenyang were higher than that in Tianjin [43]. In addition, the content of Cu was higher than that in the sewage irrigation areas in Tongzhou District, Beijing [44]. The difference in heavy metal contents between these sewage irrigation areas may be attributed to the distinctive sources of sewage, irrigation frequency, and soil background values. In the studied area, there were many manufacturing facilities, including metal casting, nonferrous metal, chemical, and leather-making industries, which were responsible for the discharge of heavy metal pollutants into the water and soil. These local industries are likely the determinant of the type and level of heavy metal pollutants in the study area. Although previous reports suggest that heavy metal pollution may also be affected by atmospheric sedimentation and traffic [18,45], the actual impact of these factors on the pollution in the study area requires further investigation.

#### 3.1.2. Spatial Distribution of the Heavy Metals in the Soil

**Figure 2:**
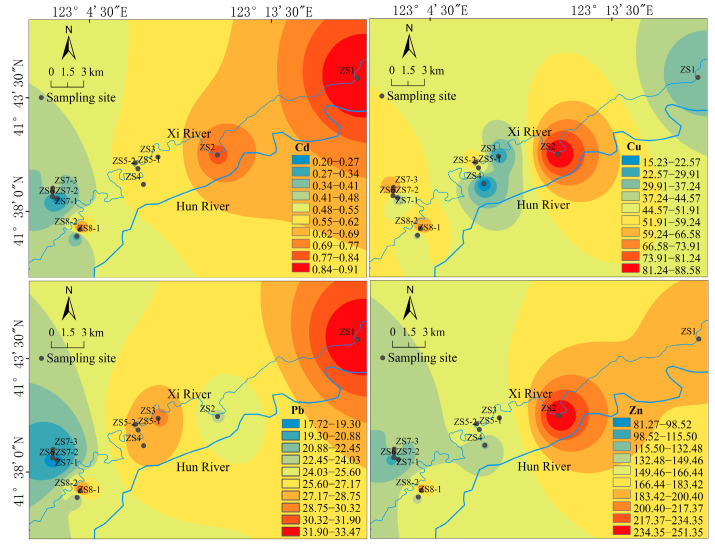
Spatial distributions of Cd, Cu, Pb, and Zn in soils of the study area (mg·kg^−1^).

The spatial distribution of the sampling points in the study area is shown in Figure 2. Cd, Cu, Pb, and Zn demonstrated different spatial distribution patterns. The overall contamination level of Cd was moderate, with the maximum values mainly distributed in the northwest part, decreasing from the northwest to the southwest along the river. The locations with the maximum levels of Cu were relatively scattered, with higher values in the central and west parts and the lower values in the north. Moreover, the concentration of Cu demonstrated a large variation, ranging from15.23 to 88.58 mg·kg^−1^. The spatial distribution of Pb was also scattered, but the pattern was opposite, with the high-value points mainly distributed in the northwest and central parts. The spatial distribution of Zn was similar to Cd, decreasing from the southeast to the northwest. The maximum value area for Zn was located in ZS2, with the minimum value in the north. In general, the concentration distribution of Zn decreased from the ZS2 point to other areas, where the concentration of Zn was mainly 150 mg·kg^−1^. Lian et al. [21] compared the concentration of heavy metals in the upstream, middle, and downstream of the Xi River sewage irrigation area and found a declining trend in the concentrations of Cd, Pb, and Zn along the river. The high level of Cd pollution in the upstream was near the Tiexi District, which was the main receiver of industrial and domestic sewage. The major sources of metal contamination in the soil were sewage irrigation and domestic and solid wastes. Although the middle and downstream of the Xi River were far away from the main pollution source, pollutant concentrations were still relatively high. This phenomenon suggests that self-purification capacity could not reduce the concentration of metals to a safe level. Finally, the use of fertilizer and livestock manure may have also led to the accumulation of heavy metals in the soil due to the fact that the middle area of the Xi River was the main agricultural area.

#### 3.1.3. Ecological Risk of Soil Heavy Metal Pollution

**Figure 3:**
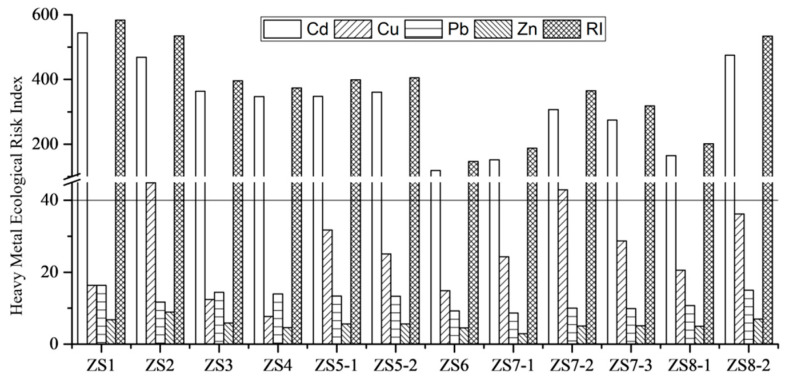
Potential ecological risk (EI) and comprehensive potential ecological risk (RI) of heavy metal elements Cd, Pb, Cu, and Zn in the soil.

The potential ecological risk of heavy metals in soil irrigated by the Xi River sewage is shown in Figure 3. The potential ecological risk factors for the four heavy metals in the soil samples demonstrated a descending order of Cd > Cu > Pb > Zn, and the potential risk factors for Cu, Pb, and Zn were all lower than the mild risk level [25]. In addition, the factors for Cd were higher than the intense risk level. Secondly, among the four metals, the contribution of Cd was the highest, and the combined potential ecological risk level varied among each sampling point. Since the study area mostly has a high ecological risk level, the resulting ecological risk issue cannot be ignored. Overall, our results showed that Cd contributed the most to the potential ecological risk, while Cu, Pb, and Zn contributed much less.

### 3.2. Heavy Metals in Vegetables

#### 3.2.1. Concentrations of Heavy Metals in Vegetables

The collected vegetables were eight leaf vegetables (i.e., cabbage, coriander, chlorella, lettuce, Chinese lettuce, asparagus leaves, celery, and spinach), one Rhizome vegetable (i.e., radish), and one Solanaceae vegetable (i.e., tomato). The moisture content of all vegetables was above 90%. The dry weights of the heavy metals in the vegetables were converted into fresh weights and compared with the safe concentration limit for Cu in “*Tolerance limit of copper in foods (GB 15199-1994)*” [46], the safe concentration limit for Zn in “*Tolerance limit of copper in foods (GB 13106-1991)*” [47], and the safe concentration limits for Cd and Pb in “*Standard of tolerance limit of pollution in food security (GB 2762-2017)*” [48], as shown in Table 3.

The concentrations of Cd, Cu, and Zn were lower than the national standard, while the level of Pb in only one sample exceeded the national standard. The concentrations of metals in Rhizome and Solanaceae vegetables were all lower than the standard values. The concentrations of Cd, Cu, and Zn in all vegetables were lower than the standard values. Except for leaf vegetables, the average concentrations of Pb in other vegetables were lower than the standard values. Considering that most vegetables are consumed in their fresh form, the dry weights of heavy metals in the vegetable samples were converted to fresh weights by the moisture coefficient.

#### 3.2.2. Accumulation of Heavy Metals

To further investigate the accumulation of heavy metals in the soil–vegetable system, the bioaccumulation factors (BCFs) for Cd, Cu, Pb, and Zn were calculated (Table 4). The average bioaccumulation factors for Cd, Cu, Pb, and Zn were in the order of Cd (0.4969) > Zn (0.1532) > Cu (0.0529) > Pb (0.0056) on the ground and Cd (0.4937) > Zn (0.1547) > Cu (0.0768) > Pb (0.0219) underground. The BCFs for Cd were higher than that of the other metals, with higher migration rates in the soil crops. The order of the bioconcentration factors (BCFs) of the heavy metals was Cd > Zn > Cu > Pb in the vegetable parts on the ground and the parts underground.

In addition, the BCFs of the four metals in different vegetables varied and demonstrated the order: leaf vegetables > root vegetable > solanaceous vegetable, which is similar to the conclusions from studies of the sewage irrigation areas in Tianjin, China [43,49]. Among the vegetables, the BCFs of leaf vegetables were the highest, indicating that this type of vegetable has a strong ability to accumulate metals in soil. This finding is similar to a report by Li et al. [50] in which leaf vegetables exhibited higher accumulation levels of heavy metals than non-leaf vegetables.

### 3.3. Health Risk Assessment

The estimated daily exposure doses are presented in Table 5, i.e., the heavy metals in males, females, and children by the four exposure pathways (i.e., direct ingestion of soil grains, inhalation, dermal contact, and dietary ingestion). Table 5 also demonstrates the differences in exposure doses for the four metals in different groups. The total daily exposure doses were in the following order: Zn > Cu > Pb > Cd for all groups. Meanwhile, by comparing the four exposure pathways, dietary ingestion was the dominant route for the total exposure doses of heavy metals (Table 5). Our data also suggest that the doses of hazardous metals in these four pathways for children are higher than that for male adults, similar to findings by Li et al. [50], Zeng et al. [51], and Chen et al. [23].The exposure doses of Cd, Pb, and Zn for female adults were higher than that of children in this study, in contrast to the results of Lian et al. [21]. Overall, our results suggest the necessity of considering the differences in exposure among genders and ages.

**Figure 4:**
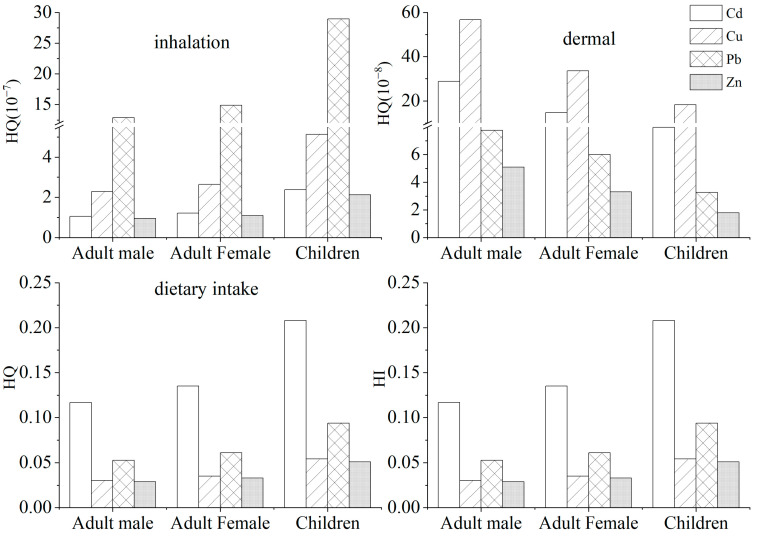
Noncarcinogenic risks of heavy metals through three exposure pathways.

Since no published reference dose (RFD)for the direct ingestion of soil grains was available, this study only included the noncarcinogenic risk index of dietary intake, inhalation ingestion, and dermal contact. The noncarcinogenic risks caused by heavy metals to children and adults in the soil and vegetable samples are presented in Figure 4. The HQ of heavy metal elements Pb and Zn demonstrated a descending order of dietary ingestion > inhalation ingestion > dermal contact, while the HQ for Cd and Cu demonstrated a descending order of dietary ingestion> dermal contact > inhalation ingestion. Similar to the daily ingestion dose, the daily ingestion dose estimated by HQs was 5–7 magnitudes higher than that of nondietary ingestion. Comparing the exposure risk by dietary ingestion of the four heavy metals, the ingestion exposure risk of Cd was the highest, which was the dominant contaminant. The HQ for Cd for male adults, female adults, and children was 0.117, 0.135, and 0.208, respectively. When comparing the exposure risks for the three routes of intake for the four elements, the highest dietary exposure risk was found for cadmium. On the one hand, cadmium was the main contaminant of noncarcinogenic risk and, on the other hand, dietary intake was the main route. Although the overall noncarcinogenic risk (HI) for all four heavy metals was less than 1, there may still be some potential health risks to the local population from dietary exposure to cadmium. According to the HI values for each heavy metal, vegetable consumption by adults and children would not cause any obvious hazard to health. In comparison, Lian et al. [21] found that Cd was the most important contaminant, and its HQ for male adults, female adults, and children was higher than the threshold value of 1, indicating harmful effects to the health of residents.

**Figure 5:**
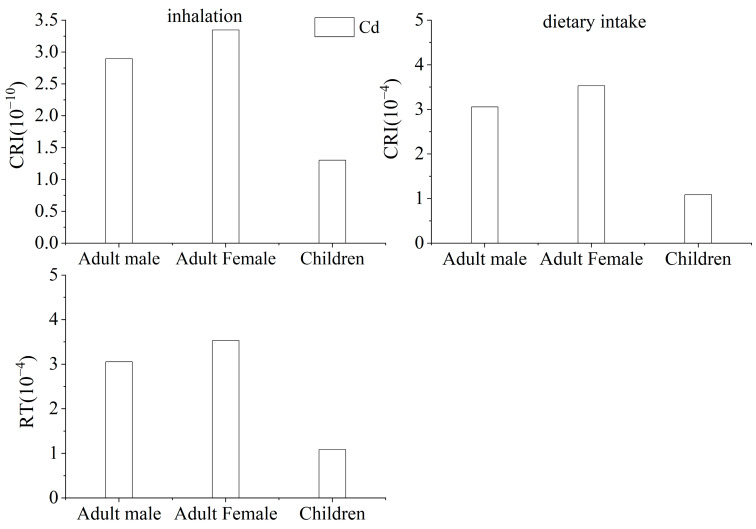
Carcinogenic risks of the heavy metals through two exposure pathways.

At present, the slope factor (SF) for the carcinogenic risk of soil intake and skin contact of Cd has not been published, which led to this study only including dietary intake and respiratory intake in the carcinogenic risk assessment, as shown in Figure 5. For male and female adults and children, the carcinogenic risk (CRI) of Cd by inhalation ingestion was 2.90 × 10^−10^, 3.35 × 10^−10^, and 1.30 × 10^−10^, respectively. The carcinogenic risk of Cd by dietary ingestion for them was 3.06 × 10^−4^, 3.53 × 10^−4^, and 1.09 × 10^−4^, respectively, which was 1.09–3.06 times higher than the suggested value of 10^−6^–10^−4^ by the US Environmental Protection Agency [27], and higher than the suggested value of 5 × 10^−5^ by the International Commission on Radiological Project (ICRP). Similar to the noncarcinogenic risk, dietary ingestion was the dominant pathway causing the estimated carcinogenic risk. In this study, dietary ingestion was the main pathway for heavy metal exposure, and the potential risk to health for all ages would increase once heavy metal pollutants were transferred into daily edible vegetables in the food chain. Further studies are needed to improve the accuracy of the model by focusing on the heavy metal exposure by dietary ingestion, to provide more detailed data on the health risk for the local community, and to provide a scientific and theoretical basis for local governments to consider adjusting agricultural production activities in the area. All this information can support the government to adjust agricultural production activities in the polluted area. The exposure risk of heavy metals to children is higher than that of adults due to the fact of several characteristics of children including eating, drinking, breathing, weight parameters lower than that of adults, and relatively weak metabolism and detoxification abilities. Hence, children are more easily affected by environmental hazards than adults [51,52,53]. As a result, the same doses of heavy metals may have negative effects on children’s development while being nonhazardous to adult health.

## 4. Discussion

The accumulation of four heavy metal elements in the soil of the sewage irrigation area of the Xi River occurred in varying degrees, and the content of three metals (i.e., Cd, Pb, and Zn) had a clear downward trend from the upstream to the middle and lower reaches, where the upper reaches were significantly higher than the middle and lower reaches. For Cd, the high level of upstream heavy metal pollutants was due to the proximity to the Tiexi District, which is the largest recipient of industrial wastewater and urban sewage in the city and an important source of heavy metal pollution from the same sewage irrigation, domestic waste, and solid waste soil. The area around the midstream is also the main agricultural base of Shenyang; thus, the application of fertilizers and livestock manure may also lead to the accumulation of heavy metals in the surrounding soils. Relevant scholars have also confirmed that large and long-term application of organic fertilizers and phosphorus-containing fertilizers from livestock and poultry manure has led to the serious accumulation of Cd [54,55]. In addition, greenhouse vegetables are commonly cultivated with mulch, and heat stabilizers containing Cd are added during the production of mulch, which may also contribute to the accumulation of Cd [56,57]. At the same time, studies [58] showed that vegetable plots are transformed from farmland with a history of frequent use of heavy metal-containing pesticides, which can leave a large amount of heavy metal contaminants.

The contents of all four elements in the soil in this study were higher than the background values and accumulated significantly, but the contents in the vegetables were relatively low, and only one leafy vegetable samples had Pb contents exceeding the national standard, indicating that the contents of heavy metals in vegetables were influenced by factors other than the total amount of heavy metals in the soil. Studies have shown that there are many factors affecting the uptake of heavy metals in vegetables such as soil physicochemical properties, the heavy metal content in soil, morphological distribution, biological effectiveness, vegetable varieties, planting management conditions, and spatial differences [59,60,61,62,63]. Therefore, when studying the heavy metal content in vegetables, attention should be paid to the possible effects of factors other than the heavy metal content in the soil. In addition, in most studies, the weight of heavy metals was calculated on a dry weight basis, while some researchers used fresh weight, and whether the standard should be unified is an issue that deserves in-depth study.

Meanwhile, due to the lack of specific ingestion data for soil particles and local vegetables, the calculation of the risk assessment model could lead to uncertainties in this research. For instance, seriously polluted areas may be far away from residents, which may overestimate the daily exposure doses via dietary ingestion, inhalation ingestion, and dermal contact. In addition, residents tend to reduce outside activities in the cold and hot seasons to reduce the opportunities of exposure to heavy metals, causing overestimation. As this study mainly referenced overseas exposure parameters [64,65], there may be uncertainties caused by factors related to regional and community differences, economic and social development levels, and different lifestyles. Parameters published in the past may also need to be updated to more accurately represent the current situation [28,29,30,31]. Many exposure parameters suitable for Western countries may not be suitable for Chinese communities [21], which would inevitably increase the uncertainty of the results. Hence, considering the uncertainty in the model’s parameters is necessary when interpreting the risk assessment in this study. Meanwhile, there may be other exposure pathways. For example, local residents may be exposed to heavy metals by drinking polluted underground water or ingesting Hg evaporated from the soil [51].

Although the abovementioned factors may bring some uncertainties, this study can still provide valuable scientific data for better pollution control and the planning of land use by local governments and policymakers.

## 5. Conclusions

This study investigated the accumulation of four heavy metals in the Xi River sewage irrigation area in Shenyang. The spatial distributions of the four metals were different, with the high-value area of Cu being scattered, while the concentrations of Cd, Pb, and Zn showed a declining trend from upstream to downstream of the Xi River. The levels of all heavy metals, except Pb, in the vegetable samples were lower than the limits in the national standard, with a slight difference. Considering the accumulation of heavy metals in the soil–vegetable system, the BCFs were in the order of Cd > Zn > Cu > Pb for all of the vegetables. The BCFs of the four heavy metals indifferent vegetables varied: leaf vegetables > Rhizome vegetable > Solanaceae vegetable. When total soil heavy metals were used for the risk assessment for children and adults by different routes of intake, the noncarcinogenic risks of the four elements were in safe ranges, while the carcinogenic risks of Cd was higher than the limit. Considering the strong ecological risk of Cd, protection measures should be taken to implement better pollution control and land use planning.

## Figures and Tables

**Table 1:** Reference parameter values adopted in the health risk assessment.

Factor	Unit	Definition	Value		
			Adult Male	Adult Female	Children
IngR [27]	mg·day^−1^	Ingestion rate of soil	50	50	100
InhR [27]	mg·day^−1^	Inhalation rate of soil	16	16	10.1
Intake [27]	kg·day^−1^	Vegetable (fresh weight)	0.26	0.26	0.13
CF [27]	kg·mg^−1^	Conversion factor	1 × 10^−6^	1×10^−6^	1×10^−6^
EF [27]	day·year^−1^	Exposure frequency	350	350	350
ED [27]	years	Exposure duration	30	30	6
BW [21]	kg	Body weight of the exposed individual	66.2	57.3	18.6
AT [27]	days	Averaged time (noncarcinogenic)	10,950	10,950	2190
		(carcinogenic)	25,550	25,550	25,550
SA [27]	cm^2^·day^−1^	Exposure skin surface area	2.15	1.89	0.76
AF [27]	mg·cm^−2^	Adherence factor	0.3754	0.3754	0.164
ABS [27]	—	Dermal absorption factor	0.001	0.001	0.001

**Table 2:** Descriptive statistics of the heavy metals and pH in the soil of the study area (*n* = 12, mg·kg^−1^).

	Cd	Cu	Pb	Zn	pH
Minimum	0.20	15.23	17.72	81.27	5.16
Maximum	0.91	88.58	33.47	251.35	6.78
Mean	0.55	50.29	24.99	157.27	5.83
SD	0.22	23.46	5.15	42.47	0.41
CV	40.84%	46.65%	20.59%	27.00%	7.07%
Skew	−0.11	0.28	0.09	0.63	0.92
Kurt	−0.74	−0.89	−1.33	1.64	1.89
Background value [37]	0.05	9.87	10.22	28.18	-
(HJ/T 333-2006) (pH < 6.5) [36]	0.3	50	50	200	-
2001–2005 [40]	0.37–2.4	16.0–52.0	30.4–127	67.8–123	-
2006–2010 [41]	0.081–0.956	22.1–40.8	31.1–86.9	-	-
2010–2014 [42]	0.005–0.88	17.14–49.89	4.13–49.25	32.93–197.85	-
Tianjin [43]	0.08–0.82	9.94–35.12	2.81–19.08	60.01–333.60	-
Beijing [44]	0.41–1.71	21.5–48.7	47.7–52.6	136–176	

**Table 3:** Concentrations of heavy metals in the vegetable samples (*n* = 35, fresh weight, mg·kg^−1^).

Vegetable Species		Fresh Weight			
		Cd	Cu	Pb	Zn
Leaf vegetables (*n* = 25)	Minimum	0.0082	0.0153	0.0007	0.4118
	Maximum	0.1575	1.5666	1.0523	8.0964
	Median	0.0254	0.2823	0.0114	2.1510
	Mean	0.0365	0.3287	0.0600	2.4880
	Limit level [46,47,48]	0.2	20	0.3	20
	RES (%)	0.00	0.00	4.00	0.00
Solanaceous vegetable (*n* = 3)	Minimum	0.0043	0.0932	0.0010	0.4882
	Maximum	0.0064	0.2299	0.0025	0.6607
	Median	0.0063	0.1357	0.0017	0.6304
	Mean	0.0056	0.1529	0.0017	0.5931
	Limit level [46,47,48]	0.05	10	0.1	20
	RES (%)	0.00%	0.00%	0.00%	0.00%
Root vegetable (*n* = 7)	Minimum	0.0062	0.1004	0.0028	0.8837
	Maximum	0.0264	0.2885	0.0727	2.7509
	Median	0.0174	0.1498	0.0054	2.0402
	Mean	0.0162	0.1861	0.0153	1.7897
	Limit level [46,47,48]	0.1	10	0.1	20
	RES (%)	0.00	0.00	0.00	0.00

RES: ratio of samples that exceeded the standard limit level.

**Table 4:** Bioaccumulation factors (BCFs) of the heavy metals in different crops (*n* = 35).

Vegetable Species		Aboveground				Underground			
		Cd	Cu	Pb	Zn	Cd	Cu	Pb	Zn
Leaf vegetables (*n* = 25)	Minimum	0.1590	0.0025	0.0003	0.0379	0.2119	0.0328	0.0050	0.0749
	Maximum	3.6859	0.1534	0.0117	1.0656	1.4025	0.3922	0.2539	0.5928
	Mean	0.8212	0.0628	0.0044	0.2009	0.7780	0.1154	0.0401	0.2150
	Median	0.5516	0.0447	0.0043	0.1620	0.7763	0.1058	0.0132	0.1847
Solanaceous vegetable (*n* = 3)	Minimum	0.1383	0.0187	0.0008	0.0330	-	-	-	-
	Maximum	0.2081	0.0441	0.0021	0.0787	-	-	-	-
	Mean	0.1625	0.0300	0.0015	0.0623	-	-	-	-
	Median	0.1412	0.0272	0.0015	0.0751	-	-	-	-
Root vegetable (*n* = 7)	Minimum	0.4162	0.0258	0.0025	0.1237	0.2062	0.0173	0.0018	0.0843
	Maximum	0.5799	0.1363	0.0331	0.2260	0.2117	0.0592	0.0058	0.1136
	Mean	0.5071	0.0661	0.0109	0.1964	0.2093	0.0382	0.0038	0.0945
	Median	0.5161	0.0362	0.0041	0.2180	0.2101	0.0382	0.0038	0.0854
Total mean		0.4969	0.0529	0.0056	0.1532	0.4937	0.0768	0.0219	0.1547

**Table 5:** Estimated exposure does (mg·kg^−1^·day^−1^) from the different exposure pathways.

	Exposure Pathway	Cd	Cu	Pb	Zn
Adult male	Ingestion	4.49 × 10^−7^	3.91 × 10^−5^	1.93 × 10^−5^	1.21 × 10^−4^
	Inhalation	1.06 × 10^−10^	9.20 × 10^−9^	4.53 × 10^−9^	2.84 × 10^−8^
	Dermal	7.23 × 10^−12^	6.29 × 10^−10^	3.10 × 10^−10^	1.95 × 10^−9^
	Dietary	1.21 × 10^−4^	1.25 × 10^−3^	3.06 × 10^−4^	8.89 × 10^−3^
	Total	1.21 × 10^−4^	1.29 × 10^−3^	3.26 × 10^−4^	9.01 × 10^−3^
Adult Female	Ingestion	5.19 × 10^−7^	4.52 × 10^−5^	2.23 × 10^−5^	1.40 × 10^−4^
	Inhalation	1.22 × 10^−10^	1.06 × 10^−8^	5.24 × 10^−9^	3.28 × 10^−8^
	Dermal	7.34 × 10^−12^	6.39 × 10^−10^	3.15 × 10^−10^	1.98 × 10^−9^
	Dietary	1.68 × 10^−3^	1.74 × 10^−2^	3.28 × 10^−3^	1.34 × 10^−1^
	Total	1.68 × 10^−3^	1.75 × 10^−2^	3.31 × 10^−3^	1.34 × 10^−1^
Children	Ingestion	3.20 × 10^−6^	2.78 × 10^−4^	1.37 × 10^−4^	8.60 × 10^−4^
	Inhalation	2.37 × 10^−10^	2.07 × 10^−8^	1.02 × 10^−8^	6.39 × 10^−8^
	Dermal	3.98 × 10^−12^	3.47 × 10^−10^	1.71 × 10^−10^	1.07 × 10^−9^
	Dietary	2.15 × 10^−4^	2.23 × 10^−3^	5.45 × 10^−4^	1.58 × 10^−2^
	Total	2.18 × 10^−4^	2.50 × 10^−3^	6.82 × 10^−4^	1.67 × 10^−2^

## Data Availability

Not applicable.
